# Randomized, Double-Blind, Pharmacokinetic Equivalence Trial Comparing DRL-Rituximab With MabThera in Patients With Diffuse Large B-Cell Lymphoma

**DOI:** 10.1200/JGO.19.00248

**Published:** 2019-12-06

**Authors:** Auro Viswabandya, Sandip Shah, Asis Mukhopadhyay, Rajnish Vasant Nagarkar, Sonica Sachdeva Batra, Luis Lopez-Lazaro, Suresh Kankanwadi, Alok Srivastava

**Affiliations:** ^1^ Christian Medical College, Vellore, India; ^2^ Vedanta Institute of Medical Sciences, Ahmedabad, India; ^3^ Netaji Subhash Chandra Bose Cancer Research Institute, Kolkata, India; ^4^ Curie Manavata Cancer Centre, Nashik, India; ^5^ Dr Reddy’s Laboratories, Hyderabad, India; ^6^ Dr Reddy’s Laboratories, Basel, Switzerland

## Abstract

**PURPOSE:**

We sought to compare the pharmacokinetics (PKs) of DRL-rituximab (DRL_RI; potential biosimilar) and innovator rituximab MabThera (Roche, Grenzach-Wyhlen, Germany; reference medicinal product [RMP]) in patients with diffuse large B-cell lymphoma (DLBCL). Efficacy, pharmacodynamics (PDs), safety, and immunogenicity were also compared.

**PATIENTS AND METHODS:**

We conducted a double-blind, parallel-group study in patients with untreated DLBCL who were eligible to receive cyclophosphamide, doxorubicin, vincristine, and prednisone (CHOP) therapy. Patients were randomly assigned at a one-to-one ratio to receive DRL_RI or RMP for six 21-day cycles of rituximab plus CHOP, with 18 months of follow-up after day 1, cycle 6 (C6). Primary end point was C1 PKs, measured as area under the plasma concentration–time curve from day 0 to 21 (AUC_0-21 days_) and maximum plasma concentration (C_max_). Equivalence was defined as 90% CIs for the DRL_RI/RMP geometric mean ratios (GMRs) within 80% and 125%. Secondary end points included efficacy noninferiority measured by objective response rate (ORR) at C6 and event-free survival and overall survival at 87 weeks, PK equivalence at C6 and PD equivalence (rate of B-cell depletion and repletion), safety, and immunogenicity. The trial was stopped after sufficient patients for primary end point evaluation were enrolled. Secondary end points are reported as observed.

**RESULTS:**

A total of 151 patients were randomly assigned (DRL_RI, n = 76; RMP, n = 75). DRL_RI/RMP GMRs for AUC_0-21 days_ and C_max_ in C1 were 99.77 (90% CI, 87.60 to 113.63) and 96.19 (90% CI, 88.65 to 104.38), respectively. ORR at C6 for DRL_RI and RMP were 82.0% and 84.8%, respectively. Rates of B-cell depletion/repletion, immunogenicity, and adverse events were comparable in both groups.

**CONCLUSION:**

DRL_RI and RMP had equivalent PKs, with comparable efficacy, PDs, safety, and immunogenicity.

## INTRODUCTION

Non-Hodgkin lymphomas (NHLs) are a heterogeneous group of malignancies arising from lymphoid tissue. NHL is the 10th most common malignancy worldwide, with an estimated incidence of 385,741 cases in 2012,^1^ further increasing over the past decade.^1,2^ Diffuse large B-cell lymphoma (DLBCL), the most common NHL subtype, is a fast-growing, aggressive form comprising up to 40% of all cases globally.^3^ Addition of rituximab to the conventional standard-of-care chemotherapy cyclophosphamide, doxorubicin, vincristine, and prednisone (CHOP) has significantly improved long-term outcome in these patients.^4-6^ However, given their high cost, innovator biologics are often not accessible to most patients worldwide. Therefore, an urgent need exists for enabling patient access to quality and affordable treatments.^7,8^ Therefore, DRL*-*rituximab (DRL_RI) is being developed as a biosimilar to the reference medicinal product (RMP) rituximab MabThera (Roche, Grenzach-Wyhlen, Germany).

The primary aim of this study was to evaluate the pharmacokinetic (PK) equivalence of DRL_RI and RMP at C1 during rituximab plus CHOP (R-CHOP) therapy. Pharmacodynamics (PDs), safety, immunogenicity, and efficacy of DRL_RI and RMP were also compared.

## PATIENTS AND METHODS

### Study Design

**FIG 1:**
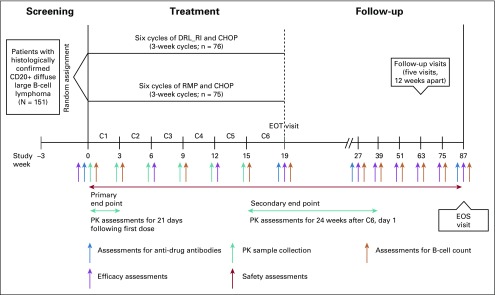
Study design. C, cycle; CD, cluster of differentiation; CHOP, cyclophosphamide, doxorubicin, vincristine, and prednisone; DRL_RI, DRL-rituximab; EOS, end of study; EOT, end of treatment; PK, pharmacokinetic; RMP, reference medicinal product.

This was a randomized, double-blind, parallel-group clinical study (Fig 1) in untreated patients with DLBCL eligible to receive R-CHOP therapy, conducted at 44 centers in India. Patients were enrolled between December 2012 and May 2015. Patients were followed-up for up to 18 months from day 1 of cycle 6 (C6). The study was approved by an independent ethics committee or institutional review board at each study center and conducted in accordance with the Declaration of Helsinki, International Council for Harmonisation Good Clinical Practice guidelines, and applicable local regulations. Each patient provided written informed consent before study entry.

After initial evaluation for eligibility verification, each enrolled patient was randomly assigned to either of the two treatment groups at a one-to-one ratio using an Interactive Voice or Web Response System. Patients were stratified based on the age-adjusted International Prognostic Index (IPI; ≤ 1 or ≥ 2).^9^ A centrally generated randomization schedule was used.

Efficacy was assessed by an independent central review of computed tomography scans. PK and PD samples were collected under standard specified conditions and analyzed at a central laboratory as per defined, validated procedures. Safety assessments were performed at an accredited central laboratory.

### Patients

Patients eligibility requirements were as follows: treatment naïve, age 18 to 60 years, diagnosed with CD20+ DLBCL (confirmed by central review of histopathology and immunochemistry), confirmed Ann Arbor/Cotswold stage II to IV disease, and adequate hepatic, renal, and bone marrow function. All inclusion and exclusion criteria are listed in the Data Supplement.

### Treatment

Patients received either intravenous DRL_RI or RMP at the approved dose of 375 mg/m^2^ body surface area^10^ over 4 hours on day 1 of each 21-day R-CHOP cycle for a total of six cycles (Fig 1). Retreatment criteria included absolute neutrophil count ≥ 1.5 × 10^9^/L (unless bone marrow was involved), platelet count ≥ 100 × 10^9^/L, appropriate liver function test results for retreatment, and nonhematologic toxicities of grade ≤ 2.

CHOP chemotherapy was administered following standard practice. Details are listed in the Data Supplement. Premedication included paracetamol, diphenhydramine or equivalent, prednisone (day-1 dose of CHOP protocol), and antiemetic prophylaxis as per institutional guidelines (Hesketh level 4 prophylaxis recommended).^11,12^ Use of prophylactic colony-stimulating factors and intrathecal methotrexate was permitted under certain circumstances (Data Supplement).

### End Points

The primary end point was comparison of area under the plasma concentration–time curve from day 0 to 21 (AUC_0-21 days_) and maximum plasma concentration (C_max_) between DRL_RI and RMP during C1, with the aim of demonstrating equivalence with the usual 80% to 125% acceptance range for the geometric mean ratio (GMR) between the test (DRL_RI) and the reference (MabThera) product.

Secondary PK end points included AUC_0-21 days_, AUC extrapolated to infinity (AUC_0-∞_), AUC to time of last quantifiable concentration (AUC_0-t_), AUC extrapolated to 24 weeks (AUC_0-24 weeks_) at C6 (analyzed using the same equivalence criteria described for C1 primary parameters), minimum plasma concentration (C_min_) and C_max_ and time of C_max_ (T_max_) at C1 and C6, half-life at C6, and predose concentration before each infusion (C_trough_).

Efficacy measured in terms of objective response rate (ORR) at C6 end-of-treatment (EOT) was to be evaluated initially using one-sided 95% CIs for the difference and an empiric noninferiority margin of −10%. EFS (event-free survival), rate of progressive disease, and OS (overall survival) at 87 weeks were also evaluated. Pharmacodynamics was evaluated in terms of B-cell depletion (defined as peripheral blood B-cell count < 20% of the lower limit of normal [LLN]; LLN, 107.0 cells/μL) and repletion (defined as ≥ 80% of baseline and above its nadir), with an empiric equivalence margin of ± 20% for the 95% CI of the difference. Other secondary end points were safety, assessed as incidence of adverse events (AEs) and serious AEs, and immunogenicity, assessed as incidence of antidrug antibodies (ADAs) and neutralizing antibodies (NAbs).

### Assessments

Complete physical examination, Eastern Cooperative Oncology Group performance status, laboratory investigations, concomitant medications, and AEs were evaluated at screening, C1, C6, EOT (week 19), follow-up visits (weeks 27, 39, 51, 63, and 75), and end-of-study (EOS; 18 months after last treatment [week 87]). During C1, PK samples were collected at preinfusion, 2 and 3 hours after beginning of infusion, end of infusion, and 0.5, 1, and 6 hours postinfusion as well as on days 2, 3, 4, 8, 15, and 22 (before C2 dosing). A preinfusion PK sample was collected for C2 to C5. During and after C6, PK samples were collected on day 1 at preinfusion, 2 hours after infusion, end of infusion, 0.5, 1, and 6 hours postinfusion, and on days 2, 4, 8, 15, and 22 as well as at weeks 4, 8, 12, and 24 from the last dose. PK parameters were estimated for patients with sufficient plasma samples, with one or more calculable values for C_max_ or AUC_0-21 days_ for C1, and patients who received six cycles of therapy and had sufficient plasma samples for C6. Samples for immunogenicity assessments were collected at screening, EOT, first follow-up (week 27), and EOS visits. Patients with positive antidrug antibodies were excluded per protocol from the main PK population. This criterion was later modified to not exclude patients from the main PK population, because the parallel design precluded biases in the evaluation resulting from this issue, and ADA-positive patient’s inclusion made the evaluation more clinically relevant. The analysis excluding these patients is included as a supportive analysis (Data Supplement). Initially it was specified that patients not meeting the steady-state criteria were to be excluded from C6 analysis, but because individual patient steady-state definitions lack robustness toward external influences, it was decided to use population-based steady-state criteria. Peripheral blood B-cell count quantification and tumor assessment details are listed in the Data Supplement.

### Statistical Analysis

Natural log-transformed primary end points were analyzed using an analysis of variance model including treatment as fixed effect. Geometric least squares mean and two-sided 90% CIs were estimated, and PK equivalence was concluded if the 90% CIs for the GMRs of C_max_ and AUC_0-21 days_ were contained within 80.00% and 125.00%.

Efficacy was evaluated using intent-to-treat (ITT; all randomly assigned patients), modified ITT (mITT; all randomly assigned patients with valid tumor assessments who received one or more doses of study drug, introduced in an amendment), and per-protocol (PP; mITT population excluding patients with major protocol deviations) populations.

The secondary end points, PDs (rates of B-cell depletion and repletion) and efficacy (ORR and the rates at 87 weeks of EFS, relapse, disease progression, and OS), were analyzed as proportion of patients within each treatment group and corresponding two-sided 95% CIs for the proportion based on the exact Clopper-Pearson method. Two-sided 95% CIs for difference in the rate of patients meeting the respective definition between groups were estimated using the Newcombe-Wilson score. Time to B-cell depletion/repletion was analyzed by the Kaplan-Meier method, with computation of CIs and *P* values by the log-rank method. For safety and immunogenicity end points, quantitative measurements were presented as descriptive statistics and qualitative measurements as frequency and percentage.

The initial sample size calculation was as follows: for the compared PK parameters in C1, we assumed a coefficient of variation (CV) of up to 50% and no difference between products. Thus, 78 evaluable patients per group would provide at least 80% power to obtain 90% CIs for their GMRs between treatments within 80% and 125%. For C6, a CV of 38% was assumed. It was planned to include 95 patients per group to account for dropouts and provide a better comparison of ORR (aiming for a noninferiority margin of −10% for the 95% CI of the difference). An unscheduled blinded sample size re-estimation was performed after 99 patients completed C1, which revealed that a sufficient number of patients had been enrolled to evaluate the primary end point, leading to the decision to stop the study. At the time of this decision, 151 patients had been enrolled. Because of this decreased sample size, the results for the other end points have been descriptively reported as observed. Details of the planned statistical analysis and sample size calculation are provided in the Data Supplement.

## RESULTS

### Patient Demographics and Baseline Characteristics

**FIG 2:**
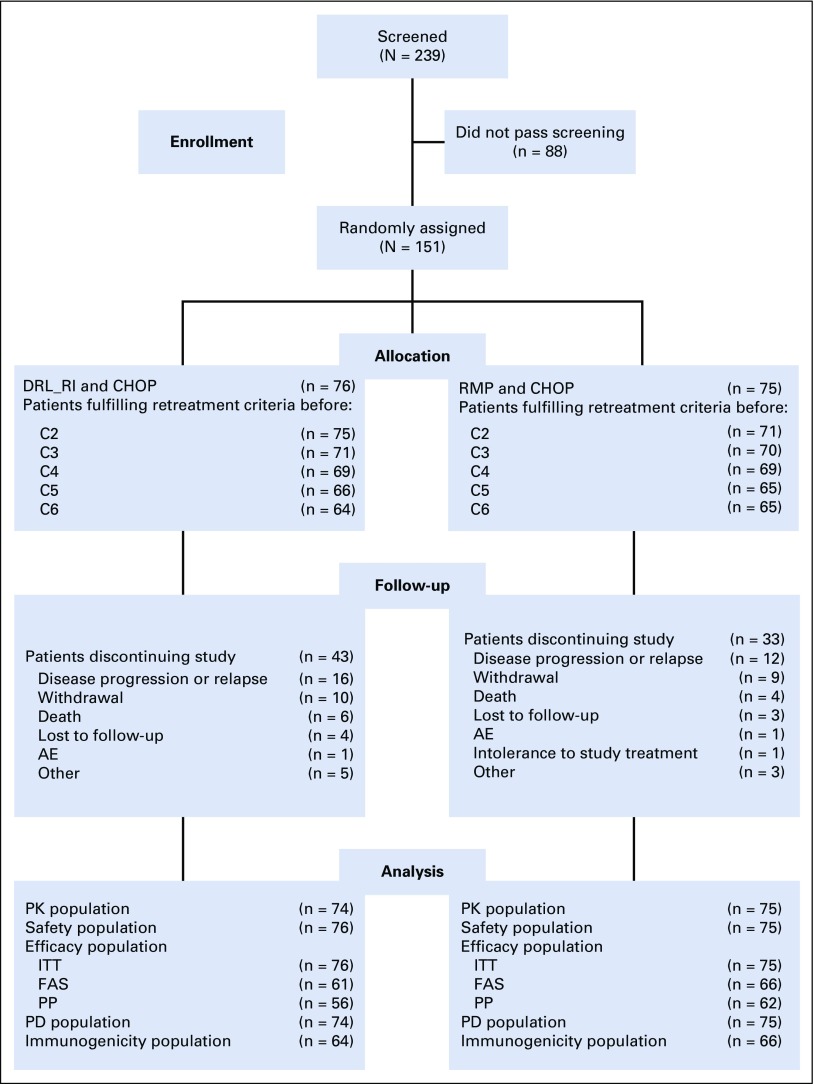
CONSORT flowchart. AE, adverse event; C, cycle; CHOP, cyclophosphamide, doxorubicin, vincristine, and prednisone; DRL_RI, DRL-rituximab; ITT, intent to treat; FAS, full analysis set; PP, per protocol; RMP, reference medicinal product.

Of the 239 patients screened, 151 were randomly assigned to receive either DRL_RI (n = 76) or RMP (n = 75), in combination with CHOP (Fig 2). Patient demographics and baseline characteristics were comparable between groups (Table 1).

**TABLE 1:**
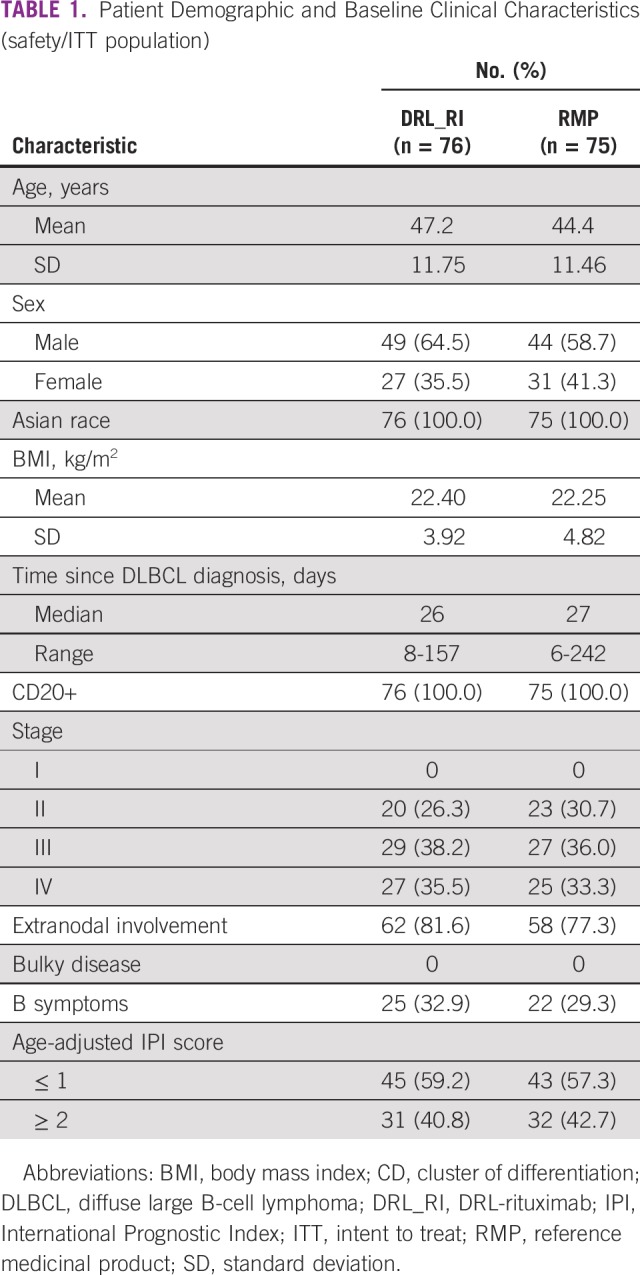
Patient Demographic and Baseline Clinical Characteristics (safety/ITT population)

### C1 PKs

**FIG 3:**
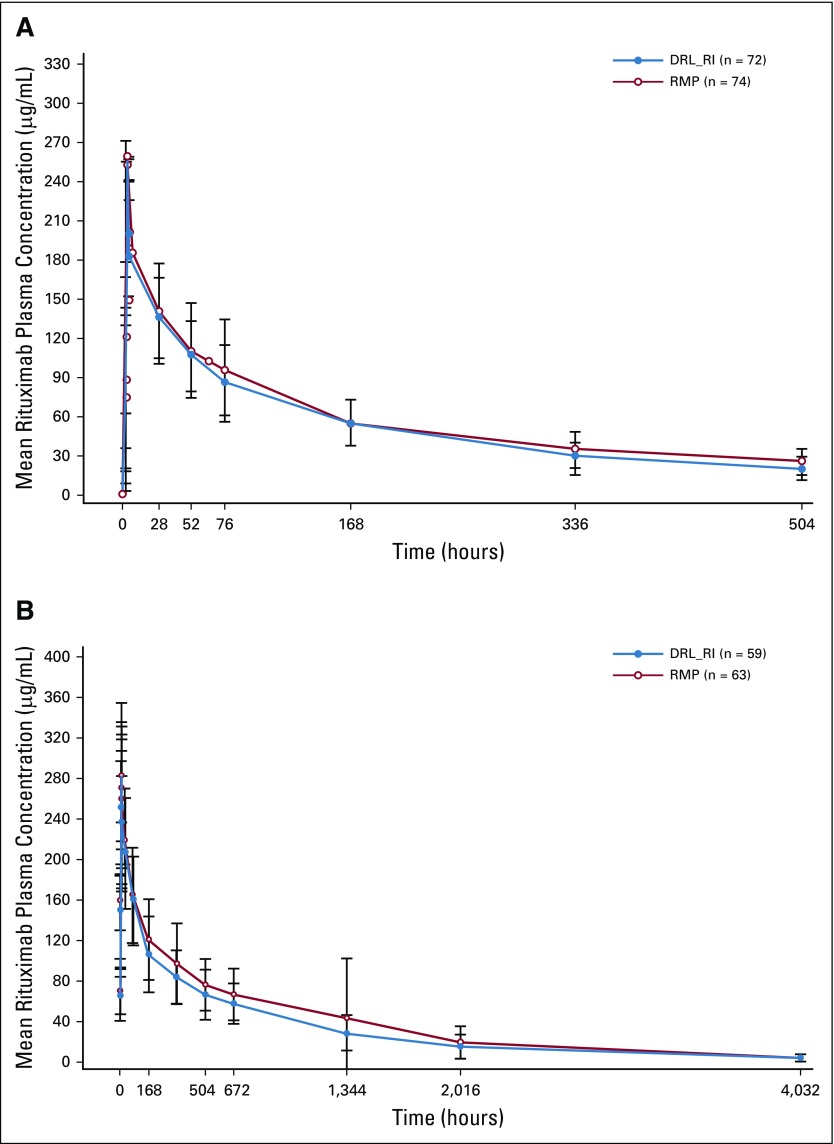
Arithmetic mean (± standard deviation) plasma concentration–time profiles for DRL-rituximab (DRL_RI) and reference medicinal product (RMP; pharmacokinetic population) in Cycles (A) 1 and (B) 6.

Mean plasma concentration–time profiles for DRL_RI and RMP in C1 were almost superimposable (Fig 3A). The 90% CIs for the GMRs of AUC_0-21 days_ and C_max_ for DRL_RI versus RMP were 99.77% (90% CI, 87.60 to 113.63) and 96.19% (90% CI, 88.65 to 104.38), respectively, establishing PK equivalence (Table 2). Geometric mean values of other PK parameters (AUC_0-t_ and C_min_) were comparable between groups (Table 2). Additional supportive (Data Supplement) and sensitivity analyses using modified PK populations revealed similar results.

**TABLE 2:**
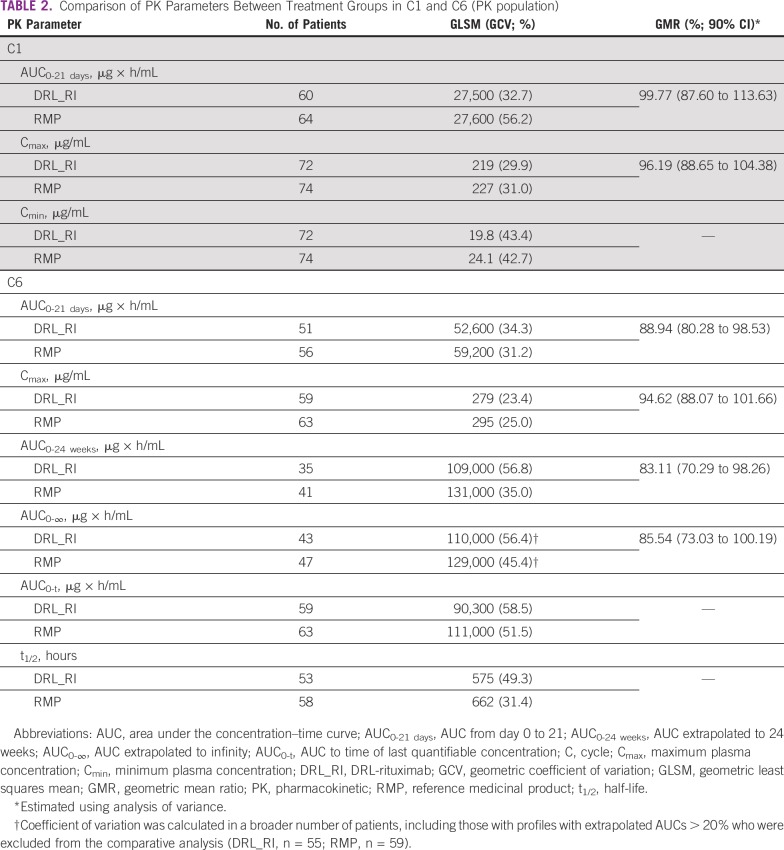
Comparison of PK Parameters Between Treatment Groups in C1 and C6 (PK population)

### Time to Steady State Evaluation

The 90% CIs of the GMRs of C_trough_ for C4 versus C5 and C6 and for C5 versus C6 were within 80% and 125%, establishing C4, C5, and C6 as steady-state cycles (Table 3).

**TABLE 3:**
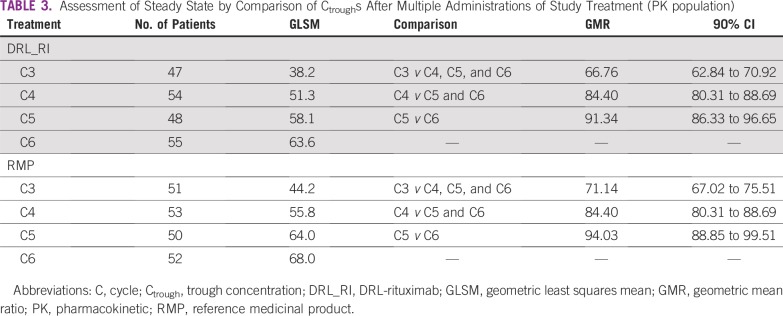
Assessment of Steady State by Comparison of C_trough_s After Multiple Administrations of Study Treatment (PK population)

### C6 PKs

Mean plasma concentration–time profiles for DRL_RI and RMP in C6 were almost superimposable (Fig 3B). Because C6 was in steady state, C6 AUC_0-21 days_ was considered the main cumulative exposure end point, in accordance with the US Food and Drug Administration guidance on clinical pharmacology end points to support demonstration of biosimilarity to a reference product.^13^ GMRs of AUC_0-21 days_ and C_max_ for DRL_RI versus RMP were 88.94% (90% CI, 80.28 to 98.53) and 94.62% (90% CI, 88.07 to 101.66), respectively (Table 2). The other PK parameters were generally comparable between groups (Table 2).

An extended PK assessment for 24 weeks after C6 (introduced in an amendment), which may be considered an exploratory evaluation after a steady-state dose,^13^ is summarized in Table 2, with some CIs extending beyond the 80% to 125% equivalence limits. Additional supportive (Data Supplement) and sensitivity analyses using modified PK populations revealed similar results, although some CIs extended below the acceptance range.

### Efficacy

Tumor response results and statistical comparisons for ORR at EOT are summarized in Table 4. At EOT, the ORRs for DRL_RI and RMP arms across all analyzed populations were as follows: 65.8% versus 74.7% in the ITT population, 82.0% versus 84.8% in the full analysis set (FAS)/mITT population, and 82.1% versus 87.1% in the PP population. The ORR with 95% CIs for the difference in the FAS/mITT population was 82.0% (95% CI, 70.0 to 90.6) for DRL_RI and 84.8% (95% CI, 73.9 to 92.5) for the RMP arm, with a difference of −2.88% (95% CI, −20.25 to 14.41). No statistically significant differences in EFS, relapsed or progressive disease rates at 87 weeks in the mITT population, or OS rate in the ITT population were observed between treatment groups (Table 5).

**TABLE 4:**
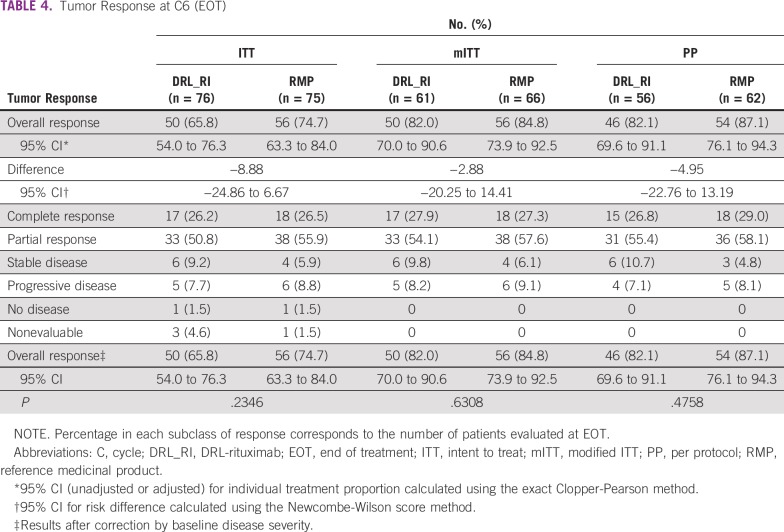
Tumor Response at C6 (EOT)

**TABLE 5:**
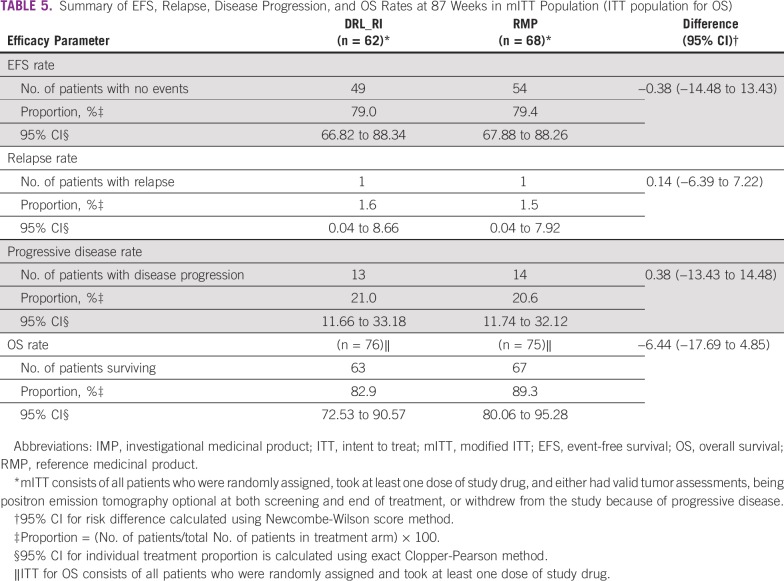
Summary of EFS, Relapse, Disease Progression, and OS Rates at 87 Weeks in mITT Population (ITT population for OS)

### PDs

At the end of C1, B-cell depletion was observed in 98.2% and 98.4% of patients in the DRL_RI and RMP arms, respectively. The difference between DRL_RI and RMP was −0.14% (95% CI, −18.03 to 17.86). B-cell repletion at the end of the follow-up period of up to 18 months was observed in 81.5% and 71.7% of patients in the DRL_RI and RMP arms, respectively. The difference between DRL_RI and RMP was 9.8% (95% CI, −8.73 to 27.76; Table 6).

**TABLE 6:**
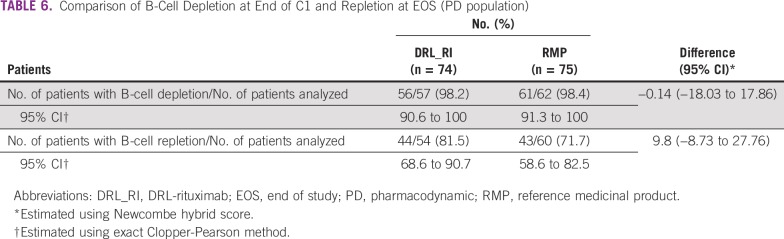
Comparison of B-Cell Depletion at End of C1 and Repletion at EOS (PD population)

Kaplan-Meier analysis of time to B-cell depletion/repletion showed a median time to B-cell depletion in both treatment arms of 2 hours (95% CI, not computable; *P* = .0733) and a median time to B-cell repletion of 9 months in both treatment arms (DRL_RI: 95% CI, 8.0 to 11.0; RMP: 95% CI, 8.0 to 9.0; *P* = .8668), with no statistically significant differences between treatments.

### Safety

A total of 2,055 treatment-emergent AEs (TEAEs) were reported (DRL_RI, n = 970; RMP, n = 1,085), of which 155 were serious TEAEs (DRL_RI, n = 74; RMP, n = 81). The most common TEAEs (≥ 20% in either/both groups) were neutropenia, anemia, leukopenia, vomiting, pyrexia, thrombocytopenia, WBC count decreased, diarrhea, hyperglycemia, febrile neutropenia, cough, and decreased weight. Table 7 shows the TEAEs with CTCAE grade 3 or 4 reported for at least 2% of patients in any treatment arm. Fifty-five (72.3%) and 63 patients (84.0%) in the DRL_RI and RMP groups, respectively, had at least one TEAE of grade 3/4. Thirteen patients (DRL_RI, n = 8; RMP, n = 5) discontinued study participation because of TEAEs.

**TABLE 7:**
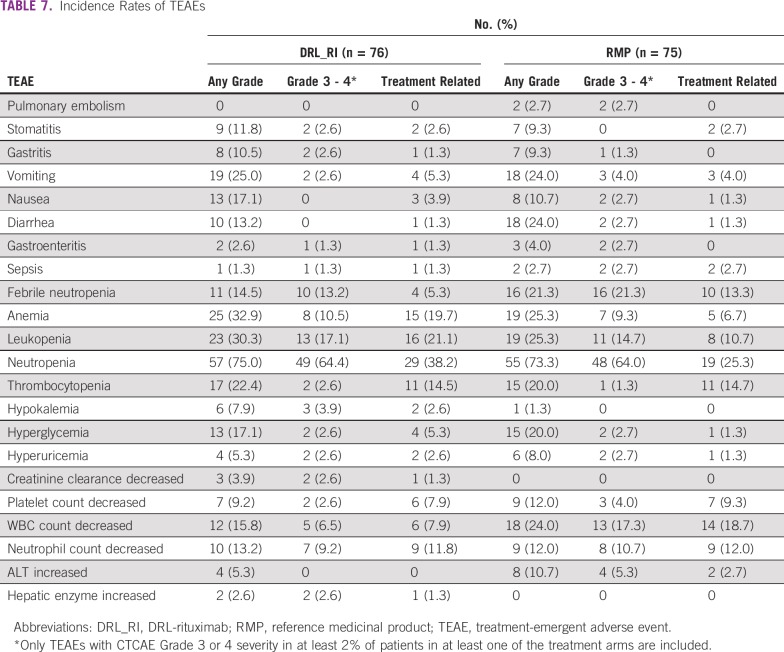
Incidence Rates of TEAEs

Nine deaths occurred up to the end of follow-up (overall rate, 6.0%). One additional death was reported after the EOS visit. Of these, three deaths were considered to be related to the investigational product by the investigators (DRL_RI, n = 2; RMP, n = 1). In one patient receiving DRL_RI, death followed febrile neutropenia with chest infection and septicemia; the febrile neutropenia was assessed by the investigator as being related to CHOP therapy and the chest infection and septicemia as being related to DRL_RI. In the other case, the cause of death was unclear because the patient died at home; it was attributed to both CHOP and DRL_RI. The third death was reported as terminal cardiac arrest, secondary to pneumonia, with septic shock associated with pancytopenia, and was considered as being related to both RMP and CHOP.

The reported causes for the deaths reported during the study considered as being unrelated to either DRL_RI or RMP were progressive disease with brain metastasis; GI hemorrhage, infection, and thrombocytopenia; myocardial infarction; death with cause not determined; probable acute cardiac event; and underlying DLBCL.

### Immunogenicity

At screening, two of 151 patients tested positive for ADAs but subsequently tested negative at all other visits. At EOS, one patient in the DRL_RI group and two patients in the RMP group developed binding ADAs but tested negative for NAbs.

## DISCUSSION

Our results show that the proposed biosimilar DRL_RI and the RMP MabThera had equivalent PK parameters after C1 as well as equivalent primary steady-state parameters AUC_0-21days_ (dosing interval) and C_max_ after C6, although for the secondary parameters at steady state AUC_0-∞_ and AUC_0-24 weeks_, confidence limits extended beyond the acceptance range.

The efficacy and B-cell depletion and repletion results were comparable for both products (Table 6). However, these comparisons could not be assessed using the original criteria, because the study enrolled fewer than the originally planned number of patients.

Comparison of the PK results of this study with those of other studies of proposed rituximab biosimilars was not possible, because those studies either evaluated patients with other indications (follicular lymphoma^14,15^ and rheumatoid arthritis^16-18^) or reported population PK analyses.^19,20^ Comparison of PK results between studies in different indications was not appropriate, because rituximab undergoes target-mediated disposition, resulting in its exposure being influenced by baseline tumor burden^21^ and changes in clearance with disease progression and response,^22^ which can vary across indications. The doses and regimens also differ for some of these indications. Results generated using population PK analysis and those generated using noncompartmental analysis are different and need to be converted using specialized methodologies, such as the ncappc R package.^23^ Previous studies do not provide conversions between population parameters and noncompartmental parameters in DLBCL,^19,20^ further precluding the possibility of such comparisons. Overall, PD results reported in terms of B-cell depletion/repletion of other studies of potential biosimilars were comparable with those of this study.^15,16,18,19^

The ORR results reported in this study are in line with the known ORRs in patients with DLBCL.^24,25^ Other reports of proposed rituximab biosimilar studies in patients with DLBCL have not included efficacy evaluations.^19,20^ At the 87-week end point, DRL_RI– and RMP-treated patients had comparable progressive disease, EFS, OS, and relapse rates.

Thus, this study presents a unique set of analyses of rituximab in patients with DLBCL, reporting multiple-dose comparative PKs of DRL_RI and the RMP MabThera, PD data on B-cell depletion/repletion, and long-term efficacy (including the 87-week EFS end point), safety, and immunogenicity data.

The original study sample size was set to ensure statistical power for its primary end point and the C6 ORR end point. However, the study was curtailed after 151 patients were enrolled (190 were planned); to achieve a complete readout of only the primary PK end point parameters of C_max_ and AUC_0-21days_ and only in C1, resulting in a smaller sample size to meet the original objectives for other end points. Therefore, although the study allows for robust analysis of the primary PK end point parameters of C_max_ and AUC_0-21days_ in C1, the evaluation of other PK/PD and efficacy parameters, while not showing obvious differences between the products, cannot be considered conclusive for noninferiority or equivalence as per the originally planned analysis.

Given the need for having a similar and homogeneous population in a comparative PK setting, this study was conducted in a single country. However, there are no known population-specific peculiarities associated with rituximab and no known ethnicity influence on its PKs; therefore, the study results can be considered generalizable. In conclusion, DRL_RI was shown to be PK equivalent to RMP (MabThera) when administered in combination with CHOP chemotherapy to patients with advanced treatment-naïve DLBCL.

## Appendix

We thank the clinical study team: Dr A. Korula (Christian Medical College, Vellore, India), Dr A. Majumdar (Nightingale Hospital, Kolkata, India), Dr B. Bagal (Tata Memorial Hospital, Mumbai, India), Dr C. Haritha (Shree Krishna Hospital & Medical Research Centre, Karamsad, India), Dr C.D. Deshmukh (Deenanath Mangeshkar Hospital & Research Center, Pune, India), Dr D.R. Samanta (Acharya Harihar Regional Cancer Centre, Cuttack, India), Dr J.K. Singh (S.S. Hospital and Research Centre, Patna, India), Dr K. Srinivasan (Dr Rai Memorial Medical Centre, Chennai, India), Dr K. Viswanathan (Meenakshi Mission Hospital & Research Centre, Madurai, India), Dr K.C. Lakshmaiah (Srinivasam Cancer Care Hospitals India, Bangalore, India), Dr M. Goyal (Apex Hospital, Jaipur, India), Dr M. Jain (K.E.M. Hospital & Research Centre, Pune, India), Dr M.V.T. Krishna Mohan (Indo American Cancer Hospital & Research Institute, Hyderabad, India), Dr N. Kilara (M.S. Ramaiah Medical College & Hospital, Bangalore, India), Dr N. Reddy (Columbia Asia Referral Hospital, Bangalore, India), Dr P. Ganeshan (Cancer Institute, Chennai, India), Dr P. Patil (Manipal Hospital, Bangalore, India), Dr R.K. Grover (Delhi State Cancer Institute, Delhi, India), Dr R. Nair (Tata Medical Centre, Kolkata, India), Dr R. Singh (SMH-Curie Cancer Centre, New Delhi, India), Dr R. Singh Arora (Sujan Surgical Cancer Hospital, Amravati, India), Dr S. Bondarde (Shatabdi Superspeciality Hospital, Nasik, India), Dr S. Damodar (Narayana Hrudayalaya Hospital, Bangalore, India), Dr S. Apte (Sahyadri Speciality Hospital, Pune, India), Dr S. Gowda Patil (HCG Bangalore Institute of Oncology, Bangalore, India), Dr S. Sinha (MNJ Institute of Oncology & Regional Cancer Centre, Hyderabad, India), Dr S. Subramanian (V.S. Hospital, Chennai, India), Dr M. Meenakshisundaram (Dr Rai Memorial Medical Centre), Dr S.V.S.S. Prasad (Apollo Hospital, Hyderabad, India), Dr V.P. Gangadharan (Lakeshore Hospital & Research Centre, Kochi, India), and Dr V. Ramanan (Jehangir Clinical Development Centre, Pune, India).
